# Mediators of weight loss in the 'Healthy Dads, Healthy Kids' pilot study for overweight fathers

**DOI:** 10.1186/1479-5868-9-45

**Published:** 2012-04-18

**Authors:** David R Lubans, Philip J Morgan, Clare E Collins, Anthony D Okely, Tracy Burrows, Robin Callister

**Affiliations:** 1 School of Education, Faculty of Education and Arts, University of Newcastle, Newcastle, NSW, 2308, Australia; 2 School of Health Sciences, Faculty of Health, University of Newcastle, Newcastle, NSW 2308, Australia; 3 Interdisciplinary Educational Research Institute, University of Wollongong, Wollongong, NSW 2522, Australia; 4 School of Biomedical Sciences and Pharmacy, Faculty of Health, University of Newcastle, Newcastle, NSW 2308, Australia; 5 Priority Research Centre in Physical Activity and Nutrition, University of Newcastle, Newcastle, Australia

**Keywords:** Physical activity, Mediation, Diet, Nutrition, Weigh loss, Intervention

## Abstract

**Background:**

A poor understanding of the specific lifestyle behaviors that result in weight loss has hindered the development of effective interventions. The aim of this paper was to identify potential behavioral mediators of weight loss in the Healthy Dads, Healthy Kids (HDHK) intervention for overweight fathers.

**Findings:**

The three-month intervention was evaluated in a randomized controlled trial and conducted in Newcastle, New South Wales, Australia. Baseline, three month (immediate post-intervention) and six month assessments were conducted. Recruitment and follow-up occurred between October 2008 and May 2009. The study sample included 53 overweight/obese men [mean ( SD) age=40.6( 97.1) years; body mass index (BMI)=33.2 (3.9) kgm^-2^] and their primary school-aged children [n=71, 54% boys; age=8.2 (2.0) years] who were randomized to HDHK program or a wait-list control group. Physical activity (PA) was assessed using pedometers and dietary behaviors were measured using a validated food frequency questionnaire. The intervention resulted in significant weight loss (5.131.27kg, *P*<0.0001) and increased PA among fathers (2769750 steps/day, *P*<0.001) and their children (1486521 steps/day, *P*<0.01). Fathers PA mediated weight loss in the intervention (AB=2.31, 95% CI=4.63 to 0.67) and was responsible for 47% of the intervention effect. Changes in dietary behaviors were not statistically significant.

**Conclusions:**

PA was an important mediator of weight loss in the HDHK intervention. Encouraging overweight fathers to be more active with their children appears to be a promising strategy for obesity treatment in men.

## Findings

### Background

The prevalence of overweight and obesity is higher among adult men than women [1,2], yet men are less likely to seek weight loss advice or volunteer for weight loss studies [3,4]. Indeed a recent systematic review [5] highlighted that males are severely underrepresented and difficult to engage in weight loss trials. As such, the evidence base for effective weight loss programs that target men is limited. 

Consequently, the development and evaluation of weight loss interventions that are attractive to men are important public health priorities.

In one of the few tailored weight loss programs for men, Morgan et al. evaluated the Self-Help Exercise and Diet using Information Technology (SHED-IT) [6] program for overweight males. To provide insights into the mechanisms of behavioral change in SHED-IT, Lubans and colleagues examined the potential mediators of weight loss [7]. None of the hypothesized behaviors satisfied the criteria for mediation, but reductions in fat intake and portion size approached statistical significance. Notably, mediation analysis has emerged as an important statistical technique for providing insights into the mechanisms of change in behavioral interventions targeting energy balance [8]. Behavioral weight loss interventions are generally based on well-defined messages targeting improvements in key dietary, PA and sedentary behaviors [9] and mediation analysis can be used to determine which behaviors contributed to weight-loss.

Parental obesity is a strong predictor of childhood weight status and interventions targeting the health behaviors of the whole family are clearly warranted [10]. However, family-based interventions typically target mothers and the influence of fathers' on their childrens health behaviors is scarce in, or not detailed in the literature [11]. The influence of fathers weight status was highlighted in a recent longitudinal study which found that children with overweight or obese fathers were at a higher risk of becoming obese [12]. The Healthy Dads, Healthy Kids (HDHK) pilot study recruited overweight fathers and their children into a randomized controlled trial [13]. The intervention resulted in significant weight-loss among fathers at six month follow-up. The aim of this paper was to identify behavioral mediators of weight-loss among fathers in HDHK intervention.

## Methods

### Study design

The study design was a two-arm randomized controlled trial. The major aim of the pilot study was to evaluate the feasibility and efficacy of a program targeting overweight/obese fathers to lose weight and model healthy lifestyles [13]. Ethics approval was obtained and participants provided written informed consent. The study was designed to detect a weight-loss difference of 3kg between groups. Assuming a standard deviation of 5kg (*P*=0.05, two-sided) and based on 80% power, a sample size of 18 fathers per group was required. The study was adequately powered to detect medium-to-large mediation effects using the bias-corrected bootstrap procedure [14].

Eligible participants were overweight/obese [body mass index (BMI) 25 to 40kg/m^2^] fathers with a child aged 512years. Participants were recruited in August/September 2008 and consisted of 53 overweight/obese men and their children (27 fathers/39 children HDHK group; 26 fathers/32 children control group). The mean age (SD) of fathers was 40.6(7.1) years, the mean (SD) BMI was 33.2(3.9) kgm^-2^. The Socio-Economic Indexes for Areas (SEIFA) (scale 1=lowest to 10=highest) based on the postal code of residence was used to determine socio-economic status (SES). The majority of participants were of middle to upper SES (SES 14=6 participants, SES 58=41 participants, SES 910=6 participants). The mean (SD) age of children was 8.2 (2.0) years, including 38 (53.5%) boys.

### Intervention

The 3-month HDHK program [13] involved fathers attending eight weekly face-to-face group sessions (75 min each). Five group sessions were for fathers only and three of the sessions were practical and involved both fathers and their children. The program was based on Banduras Social Cognitive Theory (SCT) [15] and Family Systems Theory [16].

Fathers were provided with evidence-based information about weight loss and behavior change and encouraged to role model more appropriate health behaviors. Fathers were also encouraged to spend quality time with their children using PA and shared experiences in the preparation and consumption of healthy foods as the medium. The practical sessions involved fathers and their children and focused on the following: (i) fundamental movement skills, (ii) rough and tumble play (iii) health-related fitness, and (iv) fun and active games. Resources included PA and weight-loss handbooks, a program folder outlining sessions and online component. Fathers were instructed to access a free website to self-monitor weight, dietary intake and PA (pedometers), and also weighed themselves at each session.

Aside from recruiting fathers only, the HDHK program was gender-sensitized to ensure the program and the messages delivered were meaningful and appealing to men [4]. For example, the dietary messages focused on nutrition and the mathematics of weight loss as opposed to a strict diet regime while still allowing consumption of sometimes foods and drink (i.e., alcohol). The program resources also included anecdotes and weight loss strategies that men could relate to such as examples of physical activities that men commonly participate in.

### Assessments

Fathers weight was measured in light clothing, without shoes on a digital scale to 0.1kg (model CH-150kp, A&D Mercury Pty Ltd, South Australia, Australia). Height was measured to 0.1cm using a portable stadiometer (VR High Speed Counter; Harpenden/Holtain, Mentone Education Centre, Morrabin, Victoria, Australia).

PA was measured objectively using Yamax SW700 pedometers (Yamax Corporation, Kumamoto City, Japan), which have good validity (within3% of actual steps taken) and intramodel reliability ( = 0.99)[17]. Fathers and their children were asked to wear pedometers for seven consecutive days. At baseline assessments, participants were instructed on how to attach the pedometers and asked to remove the pedometers only when sleeping, when the pedometer might get wet (e.g., swimming or showering) or during contact sports. At the end of the day participants were asked to record their steps on a log sheet and record if they had removed their pedometer. The contribution of non-wear time (e.g., swimming and contact sports) to total PA was explored by examining the self-reported log sheets completed by participants [18]. However, there were no significant differences between unadjusted and adjusted step counts and because the log sheets were not completed by all participants, the unadjusted values are reported.

Dietary intake of fathers was measured using the Dietary Questionnaire for Epidemiological Studies (DQES) Version 2, Food Frequency Questionnaire (FFQ) from the Cancer Council Victoria [19]. Although limited validation studies have been conducted in men, validation of poly- and monounsaturated fatty acid intakes using plasma fatty acid concentrations generated adjusted correlation coefficients ranging from 0.38 to 0.78 [20]. Validation of fruit and vegetable intakes using plasma carotenoid concentrations gave correlation coefficients ranging from 0.28 for lycopene to 0.46 for beta-cryptoxanthin [21]. Fruit and vegetable serves/day, alcohol consumption (% of total daily energy intake), fat intake (% of total daily energy intake), consumption of energy dense snacks (g/day) (i.e. crackers, sweet biscuits, cakes, chocolate, crisps and ice cream), take away meals (g/day) and total energy intake (kcal/day) and portion size were explored as potential mediators of weight-loss in the HDHK intervention. Portion size photographs were used to calculate single portion size factor (PSF) to determine participants usual meal portion sizes.

### Statistical analyses

All analyses were conducted using the Statistical Package for the Social Sciences (SPSS for Windows version 17.0, 2008, SPSS Inc, Chicago, IL). Last observation carried forward was used for missing data (i.e. baseline or 3-month scores carried forward). Variables were adjusted for baseline values using linear regression and the residualized change scores were used in the analyses. The SPSS macro developed by Preacher and Hayes [22] was used to calculate the regression coefficients for the effect of the intervention on the potential mediators (A) and the association between changes in mediators and changes in weight (B). The program also estimates the total (C), direct (C), and indirect (AB) intervention effects, including tests of significance using bootstrap procedures which is recommended for use in small samples [14].

## Results and discussion

Eighty-three percent of the sample were retained at six months (n=44). Participants outcomes at baseline and six months post-test are reported in Table 1. The total effect of the HDHK intervention effect on fathers weight was statistically significant (C=5.13kg, *P*<0.0001).

**Table 1:** Participants values for weight, physical activity and dietary outcomes at baseline and posttest

**Variables**	**Baseline**		**Six months posttest**	
	**Control(n = 26)**	**Intervention (n = 27)**	**Control (n = 26)**	**Intervention (n = 27)**
Weight (kg)	105.0 (13.4)	106.7 (13.7)	104.9 (13.2)	101.7 (14.6)
Physical activity (steps/day)	8028 (2559)	8522 (2745)	7405 (2591)	10,538 (3777)
Childs physical activity (mean steps/day)	10,903 (2920)	11,023 (2481)	10,394 (2929)	11,980 (2909)
Portion size (PSF)^a^	1.49 (0.30)	1.59 (0.33)	1.39 (0.35)	1.40 (0.36)
Fruit (serves/day)	1.3 (1.1)	1.1 (0.9)	1.2 (0.8)	1.5 (0.9)
Vegetable (serves/day)	2.0 (1.1)	2.4 (1.1)	2.1 (1.3)	2.3 (1.0)
Alcohol (% energy/day)	9.8 (12.5)	9.8 (11.7)	9.4 (9.6)	11.6 (13.7)
Fat (% energy/day)	36.6 (5.1)	34.7 (4.8)	36.3 (4.8)	33.3 (4.0)
Energy dense snacks (g/day)	116.5 (72.0)	98.0 (60.8)	90.2 (71.7)	59.2 (50.5)
Take away meals (g/day)	138.8 (204.1)	74.5 (56.1)	88.5 (65.5)	59.8 (53.7)
Total energy (kcal/day)	3038 (984)	2808 (854)	2640 (805)	2350 (808)

*Note.* Means reported and standard deviations in brackets.

^*a*^ Portion size photographs are used to calculated a single portion size (PSF) factor is to indicate whether on average a person eats median size serves (PSF=1), more than the median (PSF>1), or less (PSF<1) and is used to scale the serve size for vegetables, meat and casseroles.

The effect of the intervention on the potential mediators and association between changes in mediators and changes in weight are provided in Table 2. After controlling for changes in the control group, the intervention effect was equal to 2769 (*P*<0.001) steps/day. In addition, there was a significant inverse association between changes in PA and weight (*P*<0.001), suggesting that men who increased their PA also reduced their weight. Consequently, increases in mens PA mediated the effects of the HDHK intervention on their weight-loss (AB=2.31, 95% CI=4.63 to 0.67). The mediated effect was found to be 47% of the intervention effect. Few studies use mediation analysis to explore whether changes in the hypothesized mediators are responsible for the observed weight-loss. Tate et al. used change score correlations to demonstrate that changes in PA were associated with weight-loss in their online weight-loss intervention [23], but they did not report if the mediated effect was statistically significant.

**Table 2:** Effect of the intervention on the potential mediators and the association between changes in mediators and changes in weight

**Hypothesized mediators**	**Direct effect of intervention on weight**		**Intervention effect on potential mediators**		**Association between potential mediators and weight**		**Mediated effect**		
	C (SE)^a^	*P* value	A (SE)^b^	*P* value	B (SE)^c^	*P* value	AB (SE)^d^	95% CI^e^	AB/(C+AB)^f^
Physical activity (steps/day)	−2.56 (1.22)	0.0402	2769 (750)	0.0006	−0.0008 (0.0002)	0.0002	−2.31 (1.06)	−4.63 to 0.67	47%
Childs activity (mean steps/day)	−4.63 (1.35)	0.0012	1486 (521)	0.0063	−0.0002 (0.0003)	0.6213	−0.25 (0.54)	−1.31 to 0.81	5%
Portion size (PSF)	−5.09 (1.28)	0.0002	−0.02 (0.10)	0.8063	1.48 (1.88)	0.4337	−0.04 (0.21)	−0.51 to 0.45	1%
Fruit (serves/day)	−5.25 (1.30)	0.0002	0.28 (0.24)	0.2550	0.44 (0.76)	0.5689	0.12 (0.33)	−0.45 to 0.97	2%
Vegetable (serves/day)	−5.13 (1.28)	0.0002	0.00 (0.30)	1.000	0.32 (0.61)	0.5992	0.00 (0.22)	−0.58 to 0.33	0%
Alcohol (% energy/day)	−4.96 (1.26)	0.0003	2.16 (3.33)	0.5207	−0.08 (0.05)	0.1452	−0.17 (0.34)	−1.04 to 0.32	3%
Fat (% energy/day)	−4.82 (1.33)	0.0007	−2.35 (1.13)	0.0429	0.13 (0.16)	0.4224	−0.31 (0.40)	−1.24 to 0.34	6%
Energy dense snacks (g/day)	−4.76 (1.30)	0.0007	−28.71 (17.13)	0.1004	0.01 (0.01)	0.2317	−0.37 (0.33)	−1.00 to 0.30	7%
Take away meals (g/day)	−4.93 (1.31)	0.0005	−24.87 (16.57)	0.1400	0.008 (0.011)	0.4711	−0.20 (0.29)	−0.87 to 0.33	4%
Total energy (kcal/day)	−4.76 (1.28)	0.0006	−1344 (953)	0.1647	0.0003 (0.0002)	0.1564	−0.37 (0.38)	−1.33 to 0.19	7%

^a^ C=unstandardized regression coefficient of the intervention predicting change in weight with mediator in the model (SE=standard error).

^b^ A=unstandardized regression coefficient of treatment condition predicting hypothesized mediators.

^c^ B=unstandardized regression coefficient of the hypothesized mediator predicting weight with treatment condition included in the model.

^d^ 95% CI=95% confidence interval; AB=product-of-coefficients estimate.

^e^ Bootstrap bias corrected 95% confidence intervals of the mediated effect.

^f^ Proportion of intervention effect that was mediated.

The HDHK program resulted in significant increases in PA among children in the intervention group (1486 steps/day, *P*<0.01). Parental modeling of PA is an important correlate of youth activity and it was hypothesized that fathers in the intervention group would spend more time being active with their children and that their childs PA would mediate their weight-loss. This hypothesis was not supported as changes in childrens PA were not associated with fathers weight-loss. This hypothesis warrants further exploration and future studies with larger samples may be necessary to detect smaller mediation effects. The majority of family-based interventions have focused on mothers [11], yet encouraging fathers to improve weight status by increasing time spent being active with their children may be a promising public health strategy.

Changes in dietary behaviors were in the hypothesized direction but were not statistically significant. For example, fathers in the intervention group increased their consumption of fruit from 1.1 to 1.5 servings from baseline to posttest, but increases were not statistically significant. Similarly, small reductions in percentage energy intake from fat were reported by fathers in the intervention group. Reducing fat intake is an efficacious strategy for inducing short-term weight-loss in overweight individuals and a recent low-fat diet trial in women found weight-loss was greatest among those who reduced fat intake over the seven year study [24]. Females have been overrepresented in previous weight-loss interventions and little is known about mechanisms of behavior change explaining clinically important weight-loss in men.

Estimates of total energy intake decreased from 2808 to 2350kcal/day among the men in the program, similar decreases were reported by participants in the control group. Social desirability bias, volunteer bias and measurement error may explain why energy intake decreased among both groups. FFQs are useful for reporting intakes at the group level, but lack the sensitivity to detect all of the modifications in dietary intake at an individual level. Consequently, our methods of dietary assessment may be underpowered and represent a worst-case scenario. Similarly, Tate and colleagues [23] found that participants in internet education and internet behavior therapy interventions both reduced calorie intake by ~500kcal/day. Although this represented a significant time effect, the group by time effect was not statistically significant. SHED-IT [6] was another weight-loss intervention reporting significant reductions in energy intake (~500kcal/day) among both weight-loss groups, but failed to identify group by time interaction effects. These findings further illustrate the importance of improving precision in the measurement of dietary outcomes.

The strengths of this study include use of an objective measure to assess PA and sophisticated statistical analysis to identify potential mediators of weight-loss. However, there are some limitations that should be noted. Dietary intake was assessed using a FFQ and the study sample was relatively small, both of which may explain our null findings in regards to dietary intake. Finally, the study did not assess all potential dietary and behavioral weight-loss mediators, such as soda consumption and reading food labels.

## Conclusions

This study provides preliminary evidence that a behaviorally-based weight management intervention that included self-monitoring and parental role modeling is a potential efficacious strategy for improving weight status in overweight men. PA was a significant mediator of weight-loss in the HDHK intervention and strategies encouraging fathers to be more active with their children may be a promising intervention strategy.

## Abbreviations

FFQ = Food frequency questionnaire; HDHK = Healthy Dads, Healthy Kids; PA = Physical activity; SHED-IT = Self-help, Exercise and Diet using Information Technology.

## Competing interests

The authors declare that they have no competing interests.

## Authors contributions

PJM, DRL, ADO, TB, CEC and RC obtained funding for the research. All authors contributed to developing the protocols and reviewing, editing, and approving the final version of the paper. The intervention was delivered by PJM, CEC, DRL and TB. DRL conducted the analysis and DRL and RC drafted the first version of the manuscript. PJM is the guarantor and accepts full responsibility for the conduct of the study and the integrity of the data. DRL is responsible for the accuracy of the data analysis. All authors read and approved the final manuscript.
